# The Minigrant Model: A Strategy to Promote Local Implementation of State Cancer Plans in Appalachian Communities

**Published:** 2011-06-15

**Authors:** Toni Herring Bounds, Jill L. Bumpus, Bruce A. Behringer

**Affiliations:** East Tennessee State University; East Tennessee State University, Johnson City, Tennessee; East Tennessee State University, Johnson City, Tennessee

## Abstract

East Tennessee State University (ETSU) was awarded a grant through an interagency agreement between the Centers for Disease Control and Prevention and the Appalachian Regional Commission to promote cancer control activities between state comprehensive cancer control (CCC) coalitions and local Appalachian communities. We invited representatives from CCC coalitions and Appalachian communities to a forum to develop a plan of action. The attendees recommended a minigrant model that uses a request for proposals (RFP) strategy to encourage CCC coalitions and Appalachian communities to collaboratively conduct forums and roundtables locally. They set criteria to guide the development of the RFPs and the agendas for the roundtables and forums that ensured new communication and collaboration between the CCC coalitions and the Appalachian communities. We established the roundtable agenda to focus on the presentation and discussion of state and local Appalachian community cancer risk, incidence, and death rates and introduction of state cancer plans. The forums had a more extensive agenda to present cancer data, describe state cancer plans, and describe successful cancer control programs in local Appalachian communities. This article describes the ETSU minigrant model that supports forums and roundtables and reports how this strategy improves cooperative partnerships between CCC coalitions and Appalachian communities in the local implementation of state cancer plans in Appalachia.

## Introduction

As defined by the Appalachian Regional Commission (ARC), the US Appalachian region consists of 420 counties in 13 states stretching from the southern tier of New York through the hill country of northern Mississippi. This region is characterized by high poverty, low educational achievement, and a scarcity of health professionals. Rural areas in the Appalachian region have higher death rates for all cancers, lung cancer, and cervical cancer compared with US rates (1-3). A study of 3 Appalachian states (West Virginia, Pennsylvania, and Kentucky) reported that the incidence of cancers of the lung, colon, rectum, and cervix were significantly higher than the nationwide rate (4). In another multistate study of Appalachian populations, the Appalachia Community Cancer Network reported lower rates of cancer screening behaviors, higher cancer incidence and death rates, and a higher proportion of late-stage cancer diagnosis among Appalachian populations (5). In 2004, Halverson et al reported that cancer death rates in Appalachian counties were higher than in non-Appalachian counties in 10 of the 13 Appalachian states (6).

In an effort to address these cancer disparities, in 2006 a grant was awarded to East Tennessee State University (ETSU) through an interagency agreement between ARC and the Centers for Disease Control and Prevention (CDC) National Comprehensive Cancer Control Program (NCCCP). NCCCP supports state comprehensive cancer control (CCC) coalitions by providing grants to states, territories, and tribal organizations. CDC encourages the CCC coalitions to promote statewide cancer control activities by implementing state cancer plans. The goal of the CDC-ARC grant is to identify strategies that will increase involvement of Appalachian communities in the local implementation of CCC activities.

In October 2007, ETSU sponsored the Community Cancer Control in Appalachia Forum to develop a plan to implement the grant. We invited approximately 30 people, including leaders from CCC coalitions from the 13 Appalachian states and representatives from Appalachian community organizations who were recognized as providing outstanding local cancer control activities by the CCC coalitions, CDC, and ARC. These partners included local cancer patient support groups, organizations providing cancer education to the local community, and individuals and groups that raised funds to provide free cancer screenings to uninsured and underinsured people.

We created an agenda for the forum to ensure that attendees participated in several group process sessions designed to meet 3 objectives: 1) provide an opportunity for state leaders, CCC coalition members, and community groups in Appalachian states to develop cancer control activities cooperatively; 2) define state Appalachian cancer disparities; and 3) identify unique Appalachian cultural place-based characteristics that influence participation in statewide CCC coalitions. Presenters at the forum introduced Appalachian cultural cancer beliefs that included a strong sense of community, a mistrust of numbers (eg, cancer rates), and an individual involvement in cancer control activities only when encouraged through personal, not organizational, relationships (7,8).

On the basis of findings from the forum, a project advisory work group that included ARC, CDC, and CCC coalition representatives recommended a minigrant approach to promote partnerships between CCC coalitions and local Appalachian community cancer organizations. From this recommendation, we developed a request for proposals (RFP) strategy to provide funding for the following activities: 1) replicate the 2007 forum in local Appalachian communities and 2) conduct a shorter, data-driven roundtable to discuss cancer data in the local Appalachian community. This approach builds on coalition theory and lessons learned in promoting collaboration between communities and external resources (9,10).

In this article, we describe the minigrant model strategy that supported forums and roundtables. These events brought together state CCC coalitions with groups and individuals in Appalachian communities to learn about state cancer plans and encourage local implementation of cancer control activities.

## The Minigrant Request for Proposals

**Box. Comparison of Forums and Roundtables to Promote Local Implementation of State Cancer Plans in Appalachian Communities**

**Table:** 

**Forums**	**Roundtables**
Prescribed agenda based on earlier regional forum	Applicant-designed agenda based on required elements
Content: Appalachian regional speaker; cancer data presentation; community best practice panel; presentation of state cancer plan; discussion of resources, challenges, and Give-Get Grid (11) to promote collaboration	Content: Appalachian cancer data presentation and discussion
Usually full or multiple days	Usually half or partial day
Maximum funding: $5,000	Maximum funding: $2,500
Written commitment and presence required from state comprehensive cancer control (CCC) coalitions	
Eligible applicants were state or substate CCC coalitions.	Eligible applicants were Appalachian community organizations such as local affiliates of national partners, and partnerships including state or regional cancer coalitions

ETSU released the RFPs for the forums and roundtables in March 2008, for a June 30 deadline, and re-released them in December 2008 on a first-come, first-served basis until all funds were allocated. The RFPs were distributed through the project advisory work group, NCCCP, CCC coalitions, consultants, the ARC Health Policy Advisory Council, and directly to all 13 states' CCC coalitions. Further circulation occurred by word of mouth. The RFPs were also posted to the project website. ETSU project staff reviewed all submitted proposals using guidelines agreed on by ETSU, NCCCP, and ARC. To receive financial support for a roundtable or forum, CCC coalitions and Appalachian community organizations were required to identify common interests, plan their cooperative events, and share minigrant resources. Forums were more formal and involved than roundtables (Box).

### Forums

The organizations eligible to respond to an RFP for resources to conduct a forum were CCC coalitions recognized by NCCCP and Appalachian community organizations that responded on behalf of the CCC coalition. The RFP application requirements were 1) a cover sheet listing the title of the proposal, the name of the primary applicant with contact information, and key partners; 2) a plan for the forum including a description of the applicant and partners; 3) a project plan to describe the process to identify, recruit, and involve representatives from successful cancer control activities in the Appalachian substate region; 4) a profile of the history of involvement with the state CCC coalition; 5) an agenda (Appendix) including a local speaker to identify perceptions of why cancer affects Appalachian residents more adversely in terms of incidence and death rates, a presentation of incidence and death rates specific for the state's Appalachian substate region, a panel of representatives from successful cancer control activities drawn from the state's Appalachian substate region, a presentation describing the state cancer plan and the history of the state's cancer coalition, and a plan to identify and discuss the barriers to participation in cancer control activities in the Appalachian substate region; 6) a budget including details and justification with a timeline; and 7) a list of anticipated outcomes using the Give-Get Grid (11) (Appendix). Minigrants provided up to $5,000 of the costs of the forum. Ten awards were available, and we received 8 applications for 9 forums, all of which were funded. Recipients included a local cancer organization, health departments, health care providers, regional development districts, local governments, regional cancer centers, affiliates of national cancer partners, and other organizations concerned about cancer.

### Roundtables

Halverson (6) presented cancer data maps at the 2007 forum that identified patterns of high death rates in the Appalachian region. Applicants were asked to focus on these data in responding to the roundtable RFP. The RFP offered to fund up to $2,500 of direct costs for roundtables. Roundtable agendas were required to facilitate presentation of the data, discuss factors that contribute to differences in the data, and generate ideas for promoting collaboration between Appalachian communities and state CCC coalitions. Eligible applicants for the roundtable RFP were local Appalachian community organizations and state CCC coalitions. Required application elements were 1) a cover sheet listing the title of the proposal, the name of the primary applicant with contact information, and a list of key partners; 2) a plan for roundtable discussion to include a description of the applicant and partners and the history of participation and involvement with CCC coalition activities, cancer data to promote discussion, a proposed agenda (Appendix), the cancer data to be discussed, the timeline and location of the event, and a list of invitees; 3) a proposed budget; and 4) a list of expected contributions and benefits. Fifteen funding awards were available. Eight applications were received, each of which was funded, resulting in a total of 19 roundtable events.

## Results: The Minigrant Forums and Roundtables

Twenty-eight events in 10 Appalachian states were conducted between September 2008 and June 2010. A total of 622 people attended these events; 82% of attendees classified themselves as Appalachian residents. Only 22% were members of the state's CCC coalition.

### Forums

Nine forums were conducted in 7 states (Table 1). State CCC coalitions (or their substate designee) partnered with Appalachian substate regional organizations to submit forum proposals. Initially, identifying an Appalachian regional partner was difficult in some cases because of lack of familiarity between the CCC coalitions and the Appalachian communities. However, once connections were made, the planning processes between CCC coalitions and community groups progressed smoothly because of their common interests in the forum. Finding this approach useful, 2 states sponsored second forums in their Appalachian substate regions, engaging different groups and topics but maintaining similar state CCC coalition representation. Kentucky's second forum focused on a different topic, and Virginia's second forum focused on the same topic but was conducted in a different area of its Appalachian substate region. The Virginia CCC coalition found the model to be so successful at bringing together state and local cancer control partners that it found other resources to replicate the forum throughout the state.

### Roundtables

Nineteen roundtables were conducted in 7 states (Table 2). The roundtable approach helped CCC coalitions to identify local partners for cancer control activities. Grantees were varied and included local affiliates of national cancer partners (eg, Susan G. Komen for the Cure, the American Cancer Society), a regional university, state NCCCP programs, a state rural health association, and substate regional CCC coalitions.

The roundtables used similar formats, but the central topics varied. Several focused on all cancers. One grantee (Susan G. Komen for the Cure affiliate) clustered 6 roundtables in 3 contiguous states to discuss breast cancer issues. In Mississippi a regional university conducted 2 consecutive roundtables; the first roundtable identified substate strategies for cancer interventions and uncovered community environmental concerns, which were pursued in the second roundtable. Kentucky sponsored 6 roundtables (1 in each Appalachian Development District) to promote an in-depth community assessment and prioritizing process. In New York, 2 substate roundtables were held with support of active substate CCC coalitions.

### Costs

The cost to conduct the forums and roundtables was low. The average billed cost of the 9 full-day forums was $2,900 (range, $1,600-$4,900). State CCC coalitions and their partners subsidized direct costs for their forums and made indirect contributions (eg, meeting space, printing). Roundtable events were typically half-day events with an average cost of $850 (range, $425-$2,500), primarily for meals, travel, and copying expenses.

### Outcomes and evaluation

We identified several approaches to evaluate the outcomes of the forums and roundtables. ETSU project staff attended nearly every forum and roundtable and recorded observations related to event logistics, completion of agenda, attendance, and collaboration of partners. We asked all attendees to complete information sheets, which were compiled to identify demographic data, sources and frequency of cancer control communications, and feedback about the forum or roundtable. Participants provided comments about the most beneficial and least helpful aspects of the event, take-home ideas, and names of new connections for local cancer control activities. ETSU project staff identified additional outcomes through post-event evaluation telephone calls to the CCC coalition leader and the primary grantee within 3 months following the forum or roundtable. We also collected copies of media coverage. Each grantee was required to submit a final report before reimbursement, from which additional outcomes were extracted. Finally, representatives from organizations sponsoring each forum and roundtable came together at a second Appalachian Cancer Forum in August 2009 to report on their events and discuss outcomes with the project advisory work group. The ETSU project staff and the attending project advisory work group members (including CDC and ARC representatives) compiled a full list of outcomes from which the following items are summarized:

- New cancer collaborations were developed between the Appalachian community organizations and CCC coalitions for future cancer control activities.
- CCC coalitions identified new members from the Appalachian community organizations.
- New dedicated resources for cancer control activities were identified in the Appalachian substate region; for example, a community college faculty member expressed interest in compiling data and hosting events.
- State cancer registry data, highlighting Appalachian substate cancer statistics and trends, were presented. These data, in conjunction with copies of the state cancer plan, will be used for presentations in the Appalachian community and for planning related cancer control activities.
- CCC coalitions decided to include cancer control activities related to their state's Appalachian substate region in future revisions to their state cancer plans.

## Minigrant Model Strengths and Limitations

The ARC-CDC interagency agreement funding, the first to focus on CCC activities in the Appalachian substate regions, had several strengths and limitations. A strength of the minigrant program was the determination that much can be accomplished with a small investment. The minigrant process offered small budgets but produced a long list of outcomes. Most events required substantially less money than was estimated in the proposals. This finding suggests that seed money can promote a successful process leading to collaboration between state CCC coalitions and Appalachian community leaders and organizations to conduct cancer control activities.

Another strength was the prescriptive forum agendas and roundtable engagement process. Several organizers reported strong approval of the established methods and reported that having these methods in place was a key aspect of their success.

As anticipated, CCC coalitions indicated an increase in membership and in speakers and participants for subsequent events. Participants from the Appalachian substate regions left the events with new information and ideas that they were able to share with their communities.

Perhaps the most important acknowledged strength of these events was the networking that occurred. We were reminded of the importance of getting people together face-to-face. Similarly, the importance of local Appalachian community cancer partners meeting state CCC coalition personnel in their own communities cannot be overstated. CCC coalition personnel had renewed respect for the disparities faced by people living in Appalachia. Holding events in recognized Appalachian sites was important.

The successful best practices that were presented at the forums were required to be from the Appalachian region. A few states were able to identify successful local programs in their substate Appalachian region, and others identified state programs that are locally implemented. Some states presented state programs that were not implemented in the Appalachian region but for which more Appalachian visibility and participation were desired. Although participation in the forums provided state and Appalachian regional visibility, states fell short of the original goal of searching for and highlighting local successful cancer control programs. Minigrant recipients who developed new partnerships in the Appalachian substate region were able to identify successful cancer control programs with which they had been unfamiliar, and we hope that this exercise improved state awareness.

The initial time frame for responding to the RFP and conducting the roundtables and forums was during the states' annual NCCCP program reporting and grant revewal submission deadlines. Programs found it difficult to respond to the RFP and subsequently conduct the roundtables and forums. However, CDC and ARC allowed a second announcement of the RFP that better accommodated the NCCCP state program schedule. For future minigrant projects, we intend to release the RFP announcement to accommodate the program deadlines of the NCCCP state programs, which will also allow the respondents to incorporate the minigrant plans into their fiscal year schedule and provide more time between the announcement and expected start dates of the events.

In addition, staff turnover in local Appalachian community organizations created difficulties with implementation of some events, even after grant awards were made. In particular, the timing of this process coincided with tightened budgets and reduced staff nationwide for many cancer organizations.

Presentation of data excites local communities, invites comparison, and focuses the question "Why are we different?" However, several roundtables did not allot sufficient time in their agendas to identify differences in local cancer prevention and treatment resources that were perceived to affect cancer outcomes. The use of data prepared from regional and state sources had mixed results. Some grantees attempted to locate more up-to-date state data but were unsuccessful. However, some states were able to analyze their state's cancer data to compare Appalachian with non-Appalachian regions for the first time. The roundtable guidelines were less prescriptive than the forum guidelines and allowed for greater local creativity in agenda development and use of state or other cancer data. This flexibility resulted in some roundtables allotting too little time for a sufficient cancer data presentation and discussion. Future efforts should more specifically require discussion and participant interaction in response to the presentation of data.

Allowing local grantees to fully manage the event did not ensure administration of evaluations. Not all evaluation forms were completed. The best return rates were at events that used small incentives or reminders, or where event staff stood at the door collecting evaluations as people left.

The CCC coalitions were able to find willing, eligible applicants to be fiscally responsible and accountable for these grants. However, there was no uniform manner in which the coalitions and their partners became applicants for the minigrants, which resulted in some delays and confusion about sponsorship. Therefore, it is important to be aware of contractual requirements when designing a minigrant process.

### Conclusion

The minigrant model using RFPs to support forums and roundtables is a successful approach to encourage understanding and cooperation between state CCC coalitions and Appalachian communities. The forums and roundtables provided a venue for a common purpose of cancer control in local Appalachian communities. This process brought cancer partners together for the benefit of the local community.

## Figures and Tables

**Table 1:** Forums to Promote Local Implementation of State Cancer Plans in Appalachian Communities

State (Location)	**Focus**	Key Partners						Date
		CR	DOH	NCI	ACS	SU	Other	
Kentucky (Renfro Valley)	General	x	x	x		x	None	9/2008
Ohio (Glouster)	General		x	x			Ohio Partners for Cancer Control, Appalachian Community Cancer Network	11/2008
Alabama (Albertville)	Hospice	x	x		x		Hospice of Marshall County, Alabama Primary Health Care Centers	11/2008
North Carolina (Black Mountain)	General		x		x		Western North Carolina Health Network	4/2009
Virginia (Big Stone Gap)	General	x	x		x	x	Mountain Laurel Cancer Support and Resource Center, Mountain Empire Older Citizens, Inc	4/2009
Kentucky (Berea)	Colorectal cancer	x	x	x		x	None	5/2009
Pennsylvania (State College)	General		x			x	Northern Appalachian Cancer Network, Office of Rural Health	12/2009
Virginia (Abingdon)	General	x	x		x	x	Johnston Memorial Cancer Center, Healthy Appalachia Institute	12/2009
Tennessee (Chattanooga)	African American faith-based						Southside Dodson Community Health Center, Servant Leadership Institute	5/2010

Abbreviations: CR, cancer registry; DOH, department of health; NCI, National Cancer Institute; ACS, American Cancer Society; SU, state university.

**Table 2:** Roundtable Discussions to Promote Local Implementation of State Cancer Plans in Appalachian Communities

**State (Location)**	Focus	Primary Applicant	Date
Mississippi (Eupora)	Environmental concerns	Regional university	9/2008
Kentucky (Somerset)	All cancer incidence and mortality	State CCC program	10/2008
Kentucky (Hazard)	All cancer incidence and mortality	State CCC program	10/2008
Kentucky (London)	All cancer incidence and mortality	State CCC program	10/2008
Kentucky (Paintsville)	All cancer incidence and mortality	State CCC program	11/2008
Mississippi (Eupora)	Environmental concerns	Regional university	11/2008
Kentucky (Morehead)	All cancer incidence and mortality	State CCC program	11/2008
Kentucky (Ashland)	All cancer incidence and mortality	State CCC program	11/2008
New York (Oneonta)	Local resources for rural counties	Regional CCC coalition	11/2008
Tennessee (Pigeon Forge)	All cancer incidence and mortality	State Rural Health Association	11/2008
Virginia (Big Stone Gap)	Breast cancer	Local Komen^a^ affiliate	1/2009
Virginia (Abingdon)	Breast cancer	Local Komen^a^ affiliate	1/2009
Tennessee (Rogersville)	Breast cancer	Local Komen^a^ affiliate	1/2009
Tennessee (Kingsport)	Breast cancer	Local Komen^a^ affiliate	1/2009
North Carolina (Linville)	Breast cancer	Local Komen^a^ affiliate	2/2009
North Carolina (Marshall)	Breast cancer	Local Komen^a^ affiliate	2/2009
New York (Olean)	Local resources for rural counties	American Cancer Society	3/2009
South Carolina (Gaffney)	Local resources for rural counties	Local cancer support organization	5/2009
Tennessee (Powell)	Tobacco	State Rural Health Association	5/2010

Abbreviation: CCC, comprehensive cancer control.

a Susan G. Komen for the Cure.

## Appendix. Required Agenda Elements* (Listed in Request for Proposals) for Forums and Roundtables to Promote Local Implementation of State Cancer Plans in Appalachian Communities

### Forums

1. Engage regional speaker to identify why cancer may be different in the Appalachian region.
2. Present Appalachian regional cancer incidence and mortality data for all cancers and multiple types of cancer.
3. Present background on comprehensive cancer control (CCC) coalition and state cancer plans
4. Engage panel on Appalachian regional best practices.
5. Identify regional cancer resource challenges and opportunities and how collaboration of CCC coalitions and local communities could address regional cancer challenges.
6. Complete Give-Get Grid (see below) as planning tool to identify potential advantages to collaboration. Additional elements may be included that address the specific needs of a state or region.

### Roundtables

1. Include agenda for the roundtable discussion meeting(s). If multiple meetings are proposed, describe your proposed process for the multiple meetings.
2. Describe your plan to present and discuss cancer data.
3. Add agenda items that help facilitate discussion about regional differences in cancer rates.
4. List location of roundtable(s) (note, roundtable must occur in region with documented differences).
5. Identify invitees to the roundtable activities. Note which invitees have confirmed their attendance.

* If your agenda does not clearly identify these elements by the topics/headings listed, please help us out by identifying them in parentheses.

### The Give-Get Grid

A simple tool for planning and evaluating contributions and benefits that can be shared to encourage and promote CCC collaboration between local and state partners (11). This model is adapted from the evaluation model developed for the Community Partnerships for Health Professions Education Program (2004).

**Table:**

	Comprehensive Cancer Control (CCC) Programs and Coalitions	Communities
"Gives"	Things that CCC programs contribute to cancer control relationships and activities in communities	Resources that communities, their volunteers, and their organizations, can contribute to local and state cancer control programs and coalitions
"Gets"	Benefits gained by state CCC programs through expanded local relationships and cancer control activities	Benefits gained by local communities through expanded relationships with state CCC programs
